# A comparative analysis of preservation techniques for the optimal molecular detection of hookworm DNA in a human fecal specimen

**DOI:** 10.1371/journal.pntd.0006130

**Published:** 2018-01-18

**Authors:** Marina Papaiakovou, Nils Pilotte, Ben Baumer, Jessica Grant, Kristjana Asbjornsdottir, Fabian Schaer, Yan Hu, Raffi Aroian, Judd Walson, Steven A. Williams

**Affiliations:** 1 Department of Biological Sciences, Smith College, Northampton, Massachusetts, United States of America; 2 DeWorm3, Natural History Museum, London, United Kingdom; 3 Molecular and Cellular Biology Program, University of Massachusetts, Amherst, Massachusetts, United States of America; 4 Program in Statistical and Data Sciences, Smith College, Northampton, Massachusetts, United States of America; 5 Department of Global Health, University of Washington, Seattle, Washington, United States of America; 6 Program in Molecular Medicine, University of Massachusetts Medical School, Worcester, Massachusetts, United States of America; University of Cambridge, UNITED KINGDOM

## Abstract

**Background:**

Proper collection and storage of fecal samples is necessary to guarantee the subsequent reliability of DNA-based soil-transmitted helminth diagnostic procedures. Previous research has examined various methods to preserve fecal samples for subsequent microscopic analysis or for subsequent determination of overall DNA yields obtained following DNA extraction. However, only limited research has focused on the preservation of soil-transmitted helminth DNA in stool samples stored at ambient temperature or maintained in a cold chain for extended periods of time.

**Methodology:**

Quantitative real-time PCR was used in this study as a measure of the effectiveness of seven commercially available products to preserve hookworm DNA over time and at different temperatures. Results were compared against “no preservative” controls and the “gold standard” of rapidly freezing samples at -20°C. The preservation methods were compared at both 4°C and at simulated tropical ambient temperature (32°C) over a period of 60 days. Evaluation of the effectiveness of each preservative was based on quantitative real-time PCR detection of target hookworm DNA.

**Conclusions:**

At 4°C there were no significant differences in DNA amplification efficiency (as measured by C_q_ values) regardless of the preservation method utilized over the 60-day period. At 32°C, preservation with FTA cards, potassium dichromate, and a silica bead two-step desiccation process proved most advantageous for minimizing C_q_ value increases, while RNA later, 95% ethanol and Paxgene also demonstrate some protective effect. These results suggest that fecal samples spiked with known concentrations of hookworm-derived egg material can remain at 4°C for 60 days in the absence of preservative, without significant degradation of the DNA target. Likewise, a variety of preservation methods can provide a measure of protection in the absence of a cold chain. As a result, other factors, such as preservative toxicity, inhibitor resistance, preservative cost, shipping requirements, sample infectivity, and labor costs should be considered when deciding upon an appropriate method for the storage of fecal specimens for subsequent PCR analysis. Balancing logistical factors and the need to preserve the target DNA, we believe that under most circumstances 95% ethanol provides the most pragmatic choice for preserving stool samples in the field.

## Author summary

Maximizing the recovery of PCR-amplifiable DNA is an important consideration in the optimization of field-ready PCR-based diagnostic techniques. Fecal specimen preservation is particularly important due to the universal use of stool for the non-invasive diagnosis of soil-transmitted helminth (STH) infections. A comprehensive and systematic assessment of methods for maintaining and preserving soil-transmitted helminth (STH) DNA, particularly from fragile eggs subjected to nuclease exposure in stool, has not been conducted previously. Through the comparative analysis of a variety of preservation techniques, the present study demonstrates that fecal samples designated for PCR-based molecular analysis maintain sample integrity for at least 60 days when stored at 4°C. While the expedited establishment of a cold chain for stool sample storage remains the best-practice procedure for downstream molecular analysis, a variety of preservatives can facilitate the extended preservation of sample quality, even under unfavorable temperatures. Factors such as preservative cost, inhibitor resistance, toxicity, availability, associated labor, sample shipping requirements and study purpose should also be considered when determining an appropriate method for fecal specimen preservation. Taking all of these factors into consideration, the use of 95% ethanol as a preservative is recommended in most situations.

## Introduction

Fecal collection provides a non-invasive sampling method for the diagnosis of intestinal parasitism [1,2]. Given the global expansion of mass deworming efforts, there is a significant need for improved surveillance that can provide sensitive and accurate diagnosis of soil-transmitted helminth (STH) infections, particularly in areas of low infection intensity and low prevalence of infection [3]. DNA-based diagnostic testing using quantitative real-time PCR provides an ideal means for the sensitive and species-specific detection of STH infection [4,5]. However, accurate PCR-based diagnosis from feces relies on the successful preservation of DNA in patient-obtained fecal material. Since immediate DNA isolation from fresh stool samples is not possible in the field, freezing samples quickly can prevent DNA degradation from the many nucleases found within feces [6]. While prompt freezing provides an optimal method for stool storage, it is impractical under field conditions in many parasite-endemic settings. As a result, stool samples are typically subjected to temperature fluctuations during collection and while in transit from remote endemic regions to a laboratory for storage and analysis. Additionally, power failures can expose specimens to increased heat, which can have a substantial impact on DNA recovery and extraction efficiency [7,8]. Given the risk that such conditions present, alternative methods for sample storage and stabilization require investigation.

Multiple studies have attempted to identify appropriate methods for the preservation of fecal material for the subsequent molecular detection of bacteria or viruses [9,10]. However, as microscopy has historically been considered the gold standard for the diagnosis of intestinal parasite infections, most STH-related studies have focused on identifying efficient stabilizing agents for the preservation of egg and larval morphology [11]. For such studies, egg hatching or egg degradation soon after shedding presents a considerable challenge with the capacity to significantly impact diagnostic accuracy [11]. Cysts from some species, such as *Dientamoeba fragilis*, and eggs from others, like *Necator americanus*, present particularly difficult challenges for detection by microscopy due to the rapid degradation of their fragile outer shells, and multiple studies have demonstrated that a significant percentage of hookworm eggs shed in the feces are degraded or damaged even in the presence of sample preservative [12,13]. Similar concerns exist for PCR-based diagnostic methods, as the breakdown of parasite eggs leads to the release of their nucleic acid content and its subsequent exposure to the various nucleases found in stool. This exposure increases the likelihood of DNA degradation [14], presenting a critical challenge, as PCR relies on the amplification of target DNA. Detection of stool specimen-derived nucleic acids is further hampered by the organic content of stool, as PCR-inhibitory substances including urates, bile salts, complex polysaccharides, bilirubin, and the byproducts of hemoglobin breakdown detrimentally impact the function of DNA polymerases required for PCR amplification [15,16]. Consequently, preservation methods most amenable to downstream molecular analysis should protect the target DNA from endogenous nucleases and chemicals that degrade DNA.

Historically, many methods have been employed for stool sample preservation. Potassium dichromate has been utilized for the downstream detection of DNA from both *Giardia* cysts and soil-transmitted helminth (STH) eggs and 10% formalin has been employed for preserving samples obtained from *Giardia duodenalis*-infected patients [16–18]. Similarly, ethanol at a concentration ≥70% has been successfully used for the preservation and downstream analysis of DNA isolated from stool samples obtained from both wild chimpanzees and humans [8,16,19–21]. Recognizing that higher concentrations of ethanol may allow for the more rapid penetration of cellular membranes and subsequent deactivation of nucleases [22], 95–96% solutions have been utilized as a preservative for molecular studies of the human, canine, and primate microbiomes as well as the detection of parasite DNA [8,23–26]. Various commercially available storage solutions have also been examined, including RNAlater [27,28] and PAXgene [29]. Other techniques have included a two-step method coupling an overnight incubation in 90% ethanol with subsequent silica-based desiccation [30], preservation using Whatman FTA cards [8,29,31], and the solitary use of silica gel beads alone [29,31]. Additionally, Formalternate has been previously evaluated for biological specimen storage, but has not been examined as a preservative of stool samples for downstream molecular analysis.

Here we report a comparative evaluation of eight different storage methods for stool. Aliquots of a human stool sample, spiked with a known quantity of *N*. *americanus* eggs, were subjected to DNA extraction and PCR amplification at various time points post-preservation, following storage at either 32°C or 4°C. In addition to evaluating recovery of amplifiable DNA, we also evaluated factors such as cost, ease of shipping, labor and the toxicity associated with each method. To the best of our knowledge, this study represents the first attempt to compare all of the aforementioned preservation methods utilizing human stool aliquots spiked with known concentrations of STH egg material.

## Methods

### Ethics statement

All hamster (*Mesocricetus auratus)* work was approved by the Institutional Animal Care and Use Committee at the University of Massachusetts Medical School (protocol A-2483). All housing and care of laboratory animals used in this study conformed with the US National Institutes of Health Guide for the Care and Use of Laboratory Animals in Research (see 18-F22) and with all requirements and regulations issued by the US Department of Agriculture (USDA), including regulations mandated by the Animal Welfare Act (Public Law 89–544, US Statutes at Large) as amended (see 18-F23).

The naïve human stool used in the study was collected from a five-year old child, at Baylor College of Medicine, who had never traveled outside of the United States at the time of collection. The process of the collection and storage of the stool, as well as the use of the stool, was explained to the child, and participation consent was provided by the child’s parent. Stool was collected and immediately stored at -20°C.

### Sample preparation

Six hundred and twenty-eight 50 mg aliquots of stool, obtained from a single uninfected human donor, were prepared for this study and were placed at -20°C for storage. Infected stool collected from multiple hamsters was pooled and the *N*. *americanus* egg count was determined by a modified McMaster method [32]. This stool was diluted in nuclease-free water such that 71.5 μl of stool suspension contained approximately 20 *N*. *americanus* eggs. For spiked sample preparation, 50 mg aliquots of naïve human stool were removed from -20°C and thawed. 71.5 μl of hamster stool suspension were then added to each 50 mg aliquot, creating spiked samples each containing approximately 400 eggs per gram (epg). Following the preparation of spiked samples, the appropriate preservative was added within one hour.

### Sample preservation and storage

**Fig 1:**
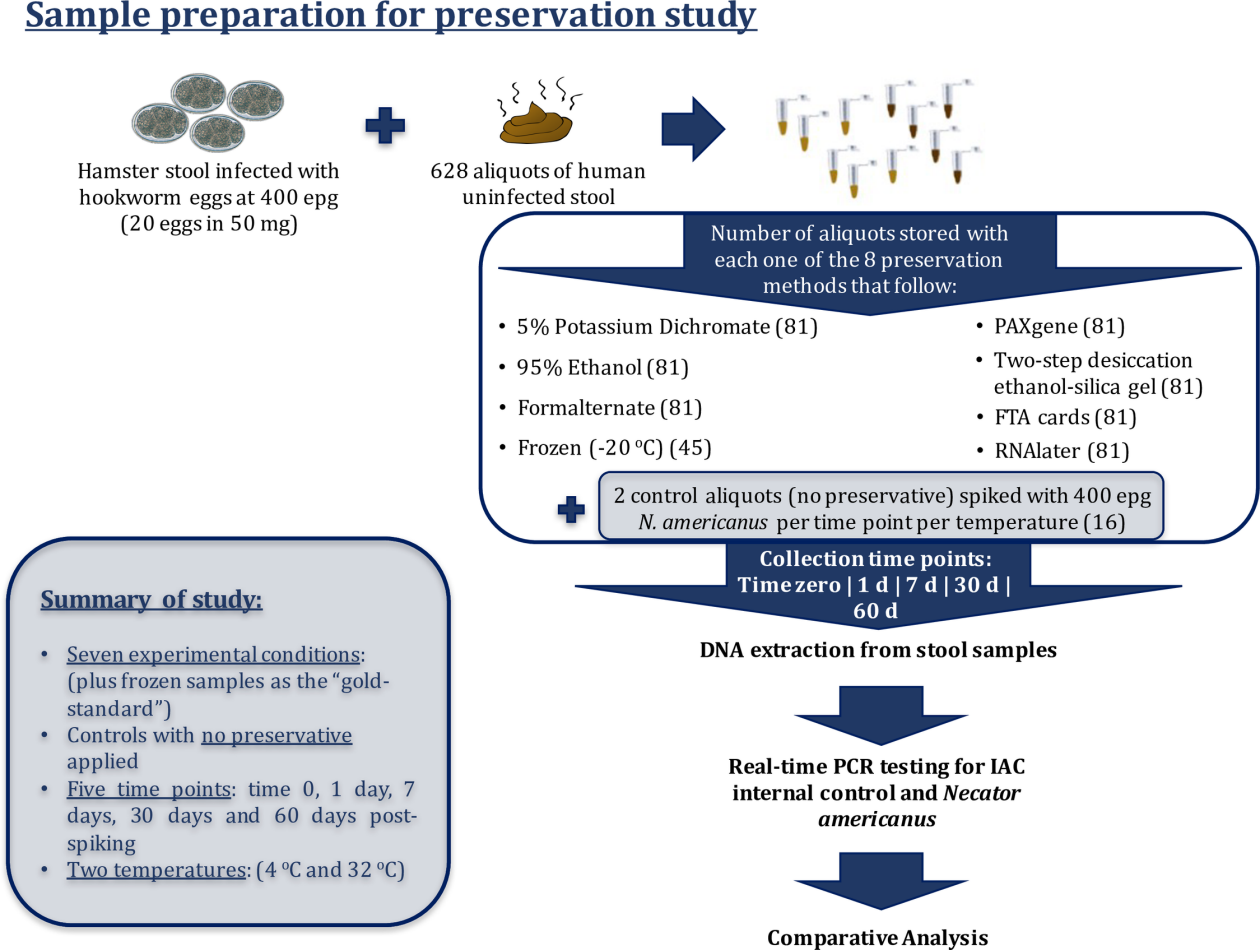
Pipeline for sample preparation. Aliquoted replicates of hamster stool with known hookworm egg counts were added to uninfected human stool in order to create a series of samples with a final concentration of 400 epg (20 eggs per 50 mg of stool). Nine spiked aliquots per preservative (for each of the seven experimental methodologies examined in the study) along with two spiked aliquots to which no preservative was added, per temperature (4°C and 32°C) and per time point (one day, seven days, 30 days, and 60 days post-spiking) were prepared. Only 45 aliquots were prepared for samples at time 0 as these samples were immediately frozen at -20°C and were therefore only exposed to a single temperature.

Samples were prepared for analysis at one, seven, 30 and 60 days post-spiking. For each time point, nine sample aliquots per preservative and per temperature were prepared as shown in Fig 1. For the time zero samples, nine sample aliquots per preservative were also prepared and, following the rapid addition and removal of preservative, each aliquot was immediately placed at -20°C until the DNA was isolated. Additionally, 45 “gold-standard” control samples that were fast-frozen at -20°C (and were therefore only subjected to a single temperature) were divided evenly for analysis among the five time points, post preservation (Fig 1). All samples prepared for storage using a given preservation technique were prepared simultaneously and were immediately transferred to either a 32°C incubator (mimicking “average tropical ambient” temperatures in the field), or to a 4°C refrigerator. The “gold standard” (-20°C) aliquots were not subjected to any supplementary preservation techniques. Two additional spiked-replicates per time-point and at each temperature were left untreated as controls.

For storage in potassium dichromate (Sigma-Aldrich, St. Louis, MO), a 5% solution was freshly prepared and added to each stool aliquot in a 1:3 mass to volume ratio. The same ratio of preservative to stool was used for storage in freshly prepared 95% ethanol (Pharmco-Aaper, Shelbyville, KY), PAXgene (PreAnalytiX, Hombrechtikon, Switzerland) and RNAlater (ThermoFisher Scientific, Waltham, MA) solutions. For storage in concentrated Formalternate (Flinn Scientific, Batavia, IL), the preservative was added in a 1:9 mass to volume ratio, in accordance with the manufacturer’s suggestions for biological specimen storage. Following the addition of the preservative, and prior to storage at the appropriate temperature, all samples were mixed vigorously using a vortex mixer.

For the two-step silica bead desiccation procedure, spiked stool samples were incubated at ambient temperature in 90% ethanol for 32 hours. Following incubation, samples were centrifuged at 20,000 x g for five minutes and the ethanol was removed. A small piece of cotton was placed between the spiked stool and approximately 700 mg of silica beads (Sigma-Aldrich, St. Louis, MO) in accordance with the manufacturer’s recommendations. When utilizing FTA cards (GE Healthcare Life Sciences, Marlborough, MA) for sample preservation, a sufficient volume of suspension was loaded onto each compartment of the card to cover the compartment’s designated surface area (approximately 125 μl per compartment). For this study, a 4-compartment card was used for 3 replicates designated for each time-point (3 cards per time point, per temperature, with one compartment on each card left empty to be used as a negative extraction control). Following the addition of the stool sample, cards were left to dry at room temperature for 2 hours before being placed into individual plastic bags with a small volume of Drierite desiccant (Hach, Loveland, CO). All cards were then transferred to 4°C or 32°C for storage.

### Removal of preservative and DNA extraction

Following preparation and preservation, all samples (except the time zero aliquots, t = 0) were incubated for one day, seven days, 30 days or 60 days at either 4°C or 32°C. After incubation, all samples (except for the FTA cards) had their preservative removed, were centrifuged at maximum speed for 5 minutes, were washed once with 1 ml of sterile water, and were transferred to -80°C until DNA extractions were performed. With the exception of samples preserved on FTA cards, DNA was extracted from each sample utilizing the entire volume of stool and using the MP Bio FastDNA SPIN Kit for Soil (MP Biomedicals, Santa Ana, CA). All extractions were performed following a slightly modified version of the manufacturer’s protocol whereby 1 μl of internal amplification control (IAC) plasmid (details below), at a concentration of 100 pg/μl, was added to each lysate just before binding the DNA in the lysate to the matrix.

In contrast, following their incubation at 4°C or 32°C, FTA cards were placed directly at -80°C. For DNA extraction from samples preserved on FTA cards, the QIAmp Micro kit (Qiagen, Hilden, Germany) was employed, and extractions were performed on the material obtained from a 6-mm diameter hole-punch per replicate. Extractions from FTA cards were performed following the manufacturer’s suggested protocol for DNA extraction from dried blood spots. The only modification to the manufacturer’s protocol was the addition of 1 μl of the IAC plasmid at a concentration of 100 pg/μl. The plasmid was added just prior to the binding of the DNA in the lysate to the column that is included in the kit.

### Internal control for efficiency of DNA extraction

An IAC plasmid, containing a unique 198-bp sequence [33] was added to each stool sample to provide a means for verification of successful DNA extraction. One microliter of the IAC control, at a concentration of 100 pg/μl, was added to all samples as described above. For detection of the IAC, the published PCR primers and probe sequences were used but with a different fluorophore and quenchers on the probe (F: 5’-CTAACCTTCGTGATGAGCAATCG-3’, R: 5’-GATCAGCTACGTGAGGTCCTAC-3’, probe:—56FAM/AGCTAGTCG/ZEN/ATGCACTCCAGTCCTCCT/3IABkFQ/-3’). Quantitative real-time PCR testing, in duplicate, was employed to verify the consistency of extraction, and to ensure that the extracted DNA was amplifiable. Both primers were used at a final reaction concentration of 250 nM and the final reaction concentration of the probe was 125 nM. Cycling conditions described previously for use with the published *N*. *americanus* assay [4], were also employed for the IAC assay. These conditions consisted of an initial 2 minutes incubation step at 50°C followed by a 10 minutes incubation at 95°C. These incubations were followed by 40 reaction cycles consisting of a 15 second denaturation step at 95°C, followed by 1 minute at 59°C for both annealing and extension. Additional details on the PCR master mix utilized in these reactions have been previously described [4].

### Column-based purification to remove PCR inhibitors

Due to the inhibitor-rich nature of biological materials found within stool, following the initial screening of a small subset of samples, all extracted samples were subjected to an additional purification step in order to reduce the presence of contaminants detrimental to downstream PCR [15]. All extracted samples, with the exception of the FTA cards, were further purified using the PowerSoil Pro Clean-up Kit (MoBio Laboratories, Inc, Carlsbad, CA) in accordance with the manufacturer’s published protocol. An additional purification step was not required for samples preserved on FTA cards, since the QIAamp DNA Micro kit, used for the FTA card extractions, already included a silica-based membrane with selective binding that facilitates the removal of contaminants/inhibitors.

### Quantitative real-time PCR testing for *N*. *americanus* DNA

For the detection of *N*. *americanus* DNA from the spiked stool samples, a previously published quantitative real-time PCR assay designed to amplify a species-specific, non-coding, tandemly repeated DNA sequence found within the genome of *N*. *americanus* was utilized [4]. All assays were performed in accordance with the published protocol, employing the published cycling conditions.

### Statistical analysis

Statistical analysis was conducted in R (R Core Team, 2017) version 3.2.3 and figures were produced using the ggplot2 package [34]. Our data consist of 628 independent observations. For ease of interpretation of the coefficients, all data were stratified by temperature. Similar to methods used in previous studies [11], a generalized linear model (GLM) [35] with a Poisson link to the C_q_ response variable as a function of the interaction between time (t, in number of days since collection) and preservation method (j) was employed. In circumstances where there was no variation in the data for a particular variable, it was omitted. Formally, we have for each temperature value:

$$\log\left(E\left[C_{q}|j,t\right]\right)=\alpha_{0}+\beta_{0}\cdot t+\alpha_{1}^{T}\cdot X_{j}+\beta_{1}^{T}\cdot X_{j}\cdot t,$$ (1)

where *X*_j_ is an indicator vector for the j^th^ treatment, the α_0_ and β_0_ coefficients are associated with the control/“no preservative” treatment, and α_1_ and β_1_ are vectors of length 8 associated with the preservation treatments (including the “immediately frozen” treatment). The α coefficients measure the change in log(C_q_) at baseline upon application, while the β coefficients measure the daily change in log(C_q_). The decision to include all terms is supported by analysis of deviance tests. The choice of a Poisson link function is motivated by the strongly right-skewed distribution of C_q_ values.

## Results

Following DNA extraction, a limited subset of the samples was tested by quantitative real-time PCR prior to undergoing additional purification by the PowerSoil Pro Clean-up Kit. Of these samples, only those previously preserved in 5% potassium dichromate demonstrated a high tolerance for the inhibitors found in feces, as demonstrated by the consistent detection of the hookworm DNA target (S1 Table).

**Fig 2:**
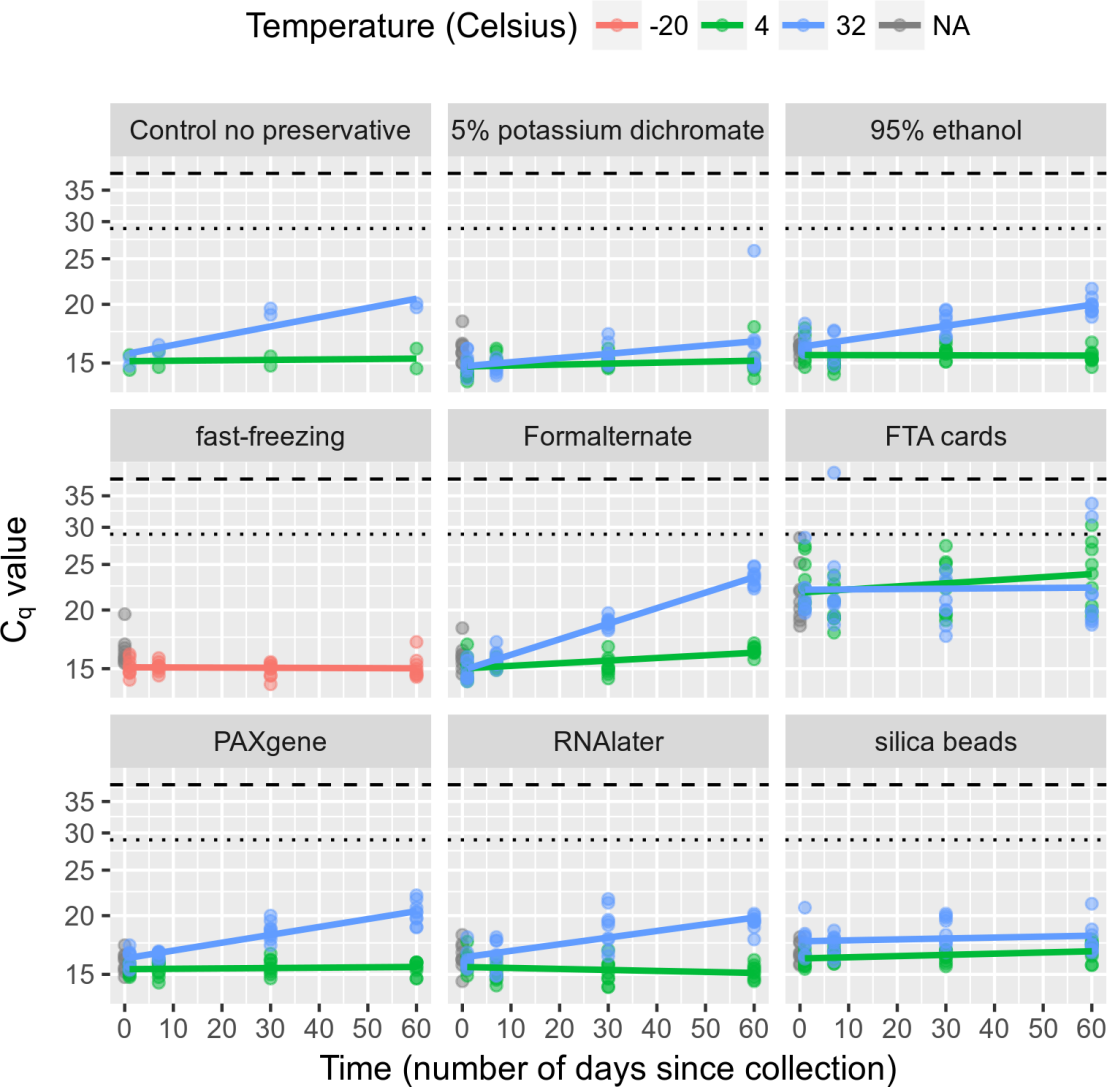
Comparison of preservation methods with Poisson GLM overlaid. Invoking the reasonable assumption of a linear relationship between log (C_q_) and time at 4°C, all tested preservation methods provided an effective means of preserving DNA for molecular analysis.

Fecal samples stored at -20°C and tested by our *N*. *americanus* quantitative real-time PCR assay, showed no significant change in C_q_ values over the 60 days of storage (F = 0.079, p = 0.7801, Fig 2, S2 Table). These data demonstrate that freezing fecal samples at -20°C in the absence of preservative is an excellent storage method for preserving DNA from *N*. *americanus* eggs. These data further confirm storage at -20°C as the “gold standard” preservation technique.

The effects of each of the eight experimental preservation methods on *N*. *americanus*-containing fecal samples stored at 4°C and 32°C over 60 days is shown in Fig 2. All of the preservation methods (including control/“no preservative”) yield similar results at 4°C with the exception of the FTA cards that showed substantially higher C_q_ values at time zero. Of note, the FTA cards were manufactured to perform best at room temperature and the volume of stool tested from the cards was less than the volume used in the other methods. All methods showed very little increase in C_q_ value over time at 4°C (S3 Table). A table with all the mean C_q_ values from the quantitative real-time PCR testing of all the biological replicates for each preservative, time point and temperature is included in the Supplementary Data (S5 Table). In contrast to the data at 4°C, at 32°C the performance of the various preservation methods showed substantial variation over time (Table 1). The deviance column in Table 1 measures the model fit where one of its three terms was deleted (time = t num_days; preservation method = X_j_ method; or the interaction of the two = t·X_j_). Higher deviance implies a poorer fit. In this model, all terms are statistically significant according to single-term deletion analysis of deviance F-tests, most importantly the interaction term (F = 11.35, p < 0.0001, see also Fig 2). The null hypothesis in each case is that the model fits just as well without that term present. Since Pr < 0.005 in each row of Table 1, we reject that null hypothesis for all three of these terms which means that it would not be appropriate to drop any of these three terms from our model.

**Table 1:** Analysis of deviance table for GLM at 32°C.

	Degrees of freedom	Deviance	F value	Pr (>F)
**<none>**		44.684		
**t, num_days**	1	46.294	8.790	0.0033
**X_j_, method**	7	81.378	28.624	0.0000
**t · X_j_, num_days: method**	7	59.237	11.353	0.0000

**Fig 3:**
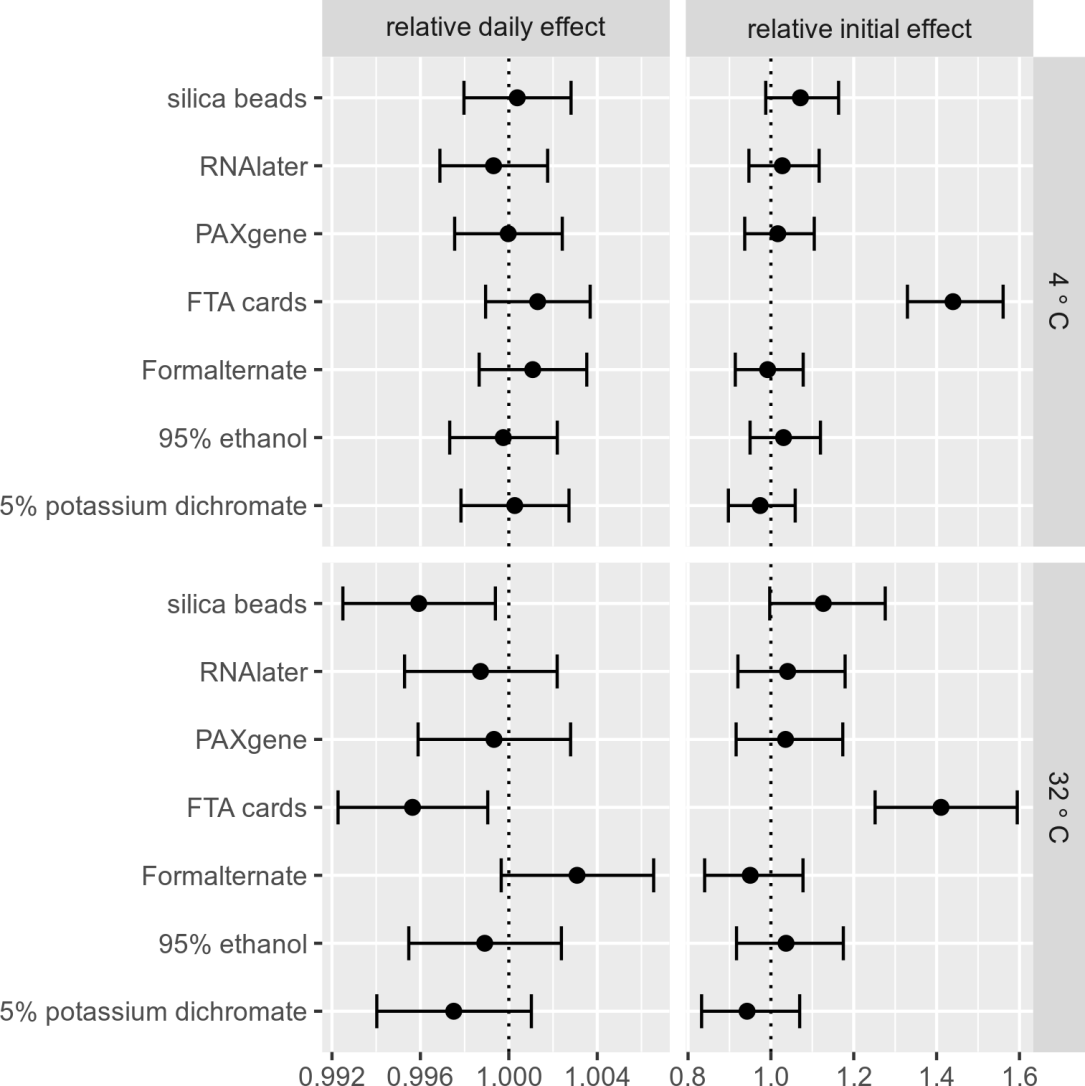
Estimates and confidence intervals for relative daily effects and relative initial effects for preservation methods at 4°C and 32°C. The graph shows the relative daily effect and the relative initial effect for each preservation method relative to the control/”no preservative” (dashed line). 5% potassium dichromate, two-step silica bead method and FTA cards seem to perform marginally better than the absence of a preservative at 32°C.

A schematic representation of the relative daily effect and relative initial effect of each preservative, at both 4°C and 32°C, is depicted in Fig 3 (a detailed description of the figure along with the coefficients, standard errors and 95% confidence intervals from the GLM model at 32°C are reported in the Supplementary Data, S4 Table). The term “relative initial effect” corresponds to the expected C_q_ difference relative to the control/”no preservative” upon application of the preservative (at time t = 0). Conversely, the term “relative daily effect” corresponds to the increase in the C_q_ value relative to the control/”no preservative” per day of storage. No significant differences among the preservatives relative to the control/“no preservative” method were observed at 4°C. Although the FTA cards start poorly due to a high initial C_q_ value (due primarily to a low amount of starting fecal material on the cards and the corresponding decreased volume of stool utilized for DNA extraction), they actually preserve the DNA most effectively at 32°C. Based on that, it seems that the FTA cards effectively offset the natural decay present in the control/ “no preservative”. However, this excellent daily preservation was not enough to offset the high starting C_q_ value and resulted in FTA cards still giving the highest C_q_ values at 60 days post-spiking (Fig 2). The second best preservation method at 32°C was the two-step silica bead method, while 5% potassium dichromate was third (performing only marginally better than the control/”no preservative”). None of the other preservation methods produced results that were statistically different from the control/”no preservative” treatment except Formalternate which performed marginally worse.

A detailed examination of the data (Table 2) shows that the C_q_ value for the control/”no preservative” increased over a 60-day time period at 32°C. Conversely, six of the seven preservation methods (all except Formalternate) reduced this increase in C_q_ value over time compared to the “no preservative” samples (i.e. Relative Daily Effect < 1, Table 2, S4 Table). The relative daily effects of RNA later, ethanol, and Paxgene are only slightly better at reducing C_q_ value increases over time compared to the untreated controls. Potassium dichromate, two-step silica bead treatment and the FTA cards were best at reducing C_q_ increases over time, while RNAlater, ethanol and Paxgene were somewhat less effective (Fig 3, Table 2).

**Table 2:** Comparison of relative daily effect sizes for the eight preservation methods at 32°C.

Preservation Method	Baseline (t = 0); exp(α_0_ + α_1j_)*	Effect; exp(β_0_ + β_1j_)^^^	Estimated C_q_ (after 60 days for each treatment)^@^	Relative Daily Effect, exp(β)^#^
**5% potassium dichromate**	14.76	1.0020	16.68	0.9975
**Silica beads– 2 step desiccation**	17.64	1.0005	18.13	0.9959
**RNA later**	16.29	1.0033	19.81	0.9987
**95% ethanol**	16.23	1.0035	19.96	0.9989
**Paxgene**	16.21	0.9995	20.45	0.9993
**Control/ “no preservative”**	15.66	1.0045	20.56	1.0000
**FTA cards**	22.09	1.0002	22.32	0.9956
**Formalternate**	14.88	1.0076	23.50	1.0031

*This column represents the estimated C_q_ at t = 0 for each preservation method.

^^^This column represents the total daily effect of treatment on the C_q_ value (that is, the C_q_ value after 1 day of storage can be obtained by multiplying the baseline value times the effect.

^@^This column represents the C_q_ value after 60 days of storage.

^#^This column represents the relative daily effect of treatment, obtained by dividing the Effect by 1.0045 (the effect value for the control). This allows us to have the control set at 1.0 for easy comparison to the other methods. Anything below 1.0 is better than the control and anything above 1.0 is worse than the control in terms of the daily effect of storage in a given preservative.

## Discussion

As a commonly tested specimen for both research-based and clinical applications, stool samples are critical to the advancement of medical understanding and to the diagnosis of many diseases. However, despite such importance, relatively little research has focused on establishing optimal techniques for the preservation of stool samples. In this study, we systematically compared the stability of DNA (as determined by the quantitative real-time PCR-based amplification of a *N*. *americanus*-specific DNA target) in stool samples using a variety of preservation techniques. Following the addition of a known concentration of hookworm-derived eggs, the DNA stability of samples preserved by immediately freezing at -20°C (the gold standard for stool sample storage) was compared with the stability of samples preserved using a variety of published techniques and commercially available DNA preservation products. This study compared DNA recovery at both 4°C and 32°C for a period of 60 days in order to simulate field conditions under “best case scenarios” (immediate refrigeration) and “worst case scenarios” (“tropical ambient temperatures”). To our knowledge, this is the first use of a quantitative real-time PCR-based assay for the monitoring of STH DNA degradation over time in similarly prepared stool samples subjected to a battery of preservation techniques at different temperatures.

Under field conditions in remote locations, specimen storage following collection can be particularly challenging. In the most extreme circumstances, specimens may face storage for days, or even weeks, at ambient temperature prior to the establishment of a cold chain. Furthermore, in resource-limited settings, a cold chain can be difficult to maintain due to unavailable, unstable or unreliable power supply, resulting in potentially damaging freeze-thaw cycles [8]. Recent work has demonstrated that refrigeration provides a viable method for up to five days of storage following the collection of fresh, untreated fecal samples, resulting in a minimal decline in egg count [11]. Since refrigeration or storage in cold boxes is often challenging in the field, we sought to determine the integrity of DNA in such samples when the benefits of 4°C storage were augmented by the addition of a variety of preservatives. Remarkably, under such conditions, all preservation methods resulted in the stable storage of stool samples at 4°C with minimal declines in PCR amplification efficiency through the point of experimental termination at day 60 post-spiking (Fig 2). These findings suggest that under field conditions, priority should be given to establishing a cold chain for sample storage as quickly as possible, since 4°C storage, even in the absence of a supplementary preservative, was sufficient to maintain sample integrity for more than seven weeks following sample collection. This finding is consistent with previous research that demonstrated the recovery of DNA from the fragile protozoan parasite *Dientamoeba fragilis* up to eight weeks, post-collection, in unpreserved stool samples [36].

In contrast, “tropical ambient” temperatures can fluctuate between 19°C and 40°C [37]. Unsurprisingly, soil-transmitted helminths have a high tolerance for heat, with egg integrity and larval survival and development possible at temperatures as high as 37°C [38]. Yet elevated temperatures also promote DNA degradation, as the activity of nucleases and other harmful enzymatic processes are increased at elevated temperatures [39]. In order to mimic the condition of samples collected in STH-prevalent regions where immediate refrigeration is not possible, we performed a parallel experiment at 32°C simulating temporary sample storage at average “tropical ambient” temperature. Under these less favorable conditions, preservation using the silica bead two-step desiccation process, 5% potassium dichromate and FTA card-based preservation appeared to have the greatest protective effects on sample integrity relative to untreated controls (as evidenced by their low “relative daily effects” on C_q_ values, Table 2). RNAlater, ethanol and Paxgene had a less protective effect on sample integrity but still performed slightly better than control/”no preservative” at all (these effects were small, however, and were not statistically significant). Preservation in Formalternate appeared to provide the least protection and in fact seemed to increase the rate of DNA degradation (Fig 3).

These data suggest that DNA from *N*. *americanus* is quite stable in stool, even in the absence of a preservative at 32°C for 10 days (Fig 2, control/”no preservative”). If storage at high temperature for more extended periods of time is anticipated before a cold chain can be established, then the two-step silica bead method or storage in 5% potassium dichromate may be the best choices for preservation. FTA cards would also constitute a viable option based on their demonstrated ability to stabilize DNA in stool over the course of time. However, additional testing will be required to optimize commercially available kits for DNA extraction from larger quantities of FTA card-preserved stool samples before the utilization of such a technique can be proposed. If storage at high temperature for extended periods is not anticipated, then storage in 95% ethanol would also be a useful option since it may provide some measure of protection against DNA degradation when compared with “no preservative” and it has the added benefit of killing many pathogens found in stool.

Of note, we understand that the results obtained in this study are based on the detection of a single STH species. However, given the vulnerability of hookworm eggs to rapid degradation in stool, we anticipate these preservation techniques will perform similarly on eggs/DNA material from other soil-transmitted helminths. Future studies should aim to verify this hypothesis.

### Conclusions

Previous work has demonstrated that in the absence of preservative, fecal egg counts are considerably reduced after a two-week period of storage at 3–5°C [11,40–42]. However, these studies have focused on the presence/absence of intact eggs for microscopy and not on the detection of intact DNA by quantitative real-time PCR assays from stool samples. In this study, stool samples maintained at the “gold standard” condition of -20°C showed no significant decrease in DNA integrity as measured by C_q_ values (S2 Table). Unfortunately, in many environments, reliable and immediate freezing is not a viable option resulting in the need for alternative approaches to sample preservation.

If conditions dictate that immediate freezing of samples is not possible, then the establishment of a 4°C cold chain provides the most effective means of preserving target DNA within a stool sample. However, given the realities of working in the field, immediate storage at -20°C or even at 4°C may not be possible. Accordingly, our results at 32°C are encouraging since the data in Fig 2 and Table 2 show that the degradation of DNA in stool samples is relatively slow upon treatment with many of the preservatives, even with storage at 32°C, for several weeks. In fact, all of the methods except Formalternate appear to provide some measure of protection. Therefore, decisions regarding the selection of a sample preservation method will most appropriately be made by considering other factors such as cost, availability of materials, toxicity, ease of use, shipping restrictions, and the labor required for sample storage.

While FTA cards provide a reliable method for DNA preservation, and can be stored in bulk at ambient temperature [8,43], they are expensive (Table 3) and the per-extraction sample mass is relatively low. Five percent potassium dichromate provides another viable option for sample preservation, demonstrating similar performance to the two-step silica bead desiccation process. However, despite successful use in field studies [16,18], the toxicity of potassium dichromate remains a significant drawback of this method, making this option logistically challenging, particularly for use in remote locations where proper disposal options are limited. Potassium dichromate is also problematic when samples need to be shipped by air, since this preservative is classified as a UN 3287, Class 6.1 Packing Group III substance, according to IATA’s (air transport) regulations.

**Table 3:** Cost estimates per sample for each preservative.

Preservative	Cost per sample ($)*
Potassium dichromate	0.0200
Fast freezing	n/a (cost of the freezer)
Formalternate	0.0022
95% ethanol	0.0025
Paxgene	0.0034
Two-step silica beads	0.0031
RNA later	0.5400
FTA cards	1.4600
Control/”no preservative”	0.0000

*cost estimates do not include labor

In contrast, while safe and effective, the two-step silica-bead desiccation method requires significantly more labor than the other tested methods. Thus, despite excellent preservation, this process presents logistical challenges, and would result in increased labor cost despite modest reagent costs. Paxgene does not show any particular advantages over the untreated control samples and Formalternate was unsuccessful in preserving stool samples, even enhancing the degradation of DNA over time (Table 2, Fig 2). Accordingly, none of these preservation methods are likely to constitute sustainable options for large field studies.

Considering all of these factors, preservation of stool samples in 95% ethanol provides the optimal balance of efficacy, safety, low cost and logistical practicality (stool samples in 95% ethanol, used as a preservative and a solution for deactivating many of the infectious agents found in stool, are classified as Biological Substance Category B, UN 3373, Class 6.2). While preservatives and techniques such as potassium dichromate, FTA cards and two-step silica desiccation may provide marginally improved preservation efficiencies when compared with 95% ethanol and therefore might be useful for select applications, significant safety hurdles, logistical difficulties and cost make these options less attractive [8,22,44,45]. Ethanol is reasonably safe, inexpensive and is readily sourced throughout most of the world. High concentrations of ethanol also result in broad viral inactivation and pathogen killing, improving safety for the laboratory workers tasked with extracting DNA from study samples.

As molecular techniques become more readily available and less expensive for the diagnosis of neglected tropical diseases, optimization of methods to improve diagnostic performance and minimize cost are increasingly important. In many settings, field-based collection of stool samples makes rapid preservation challenging, particularly the use of a cold chain. These data demonstrate that many available preservation techniques can help stabilize DNA both with and without refrigeration. However, other factors including safety concerns, cost and logistical difficulties challenge the broad implementation of many of the available techniques. Taking all factors into consideration, we believe that 95% ethanol is the best choice for use as a stool preservative under most circumstances.

## Supporting information

S1 Table Average C_q_ values from quantitative real-time PCR testing for IAC and *N*. *americanus*. Note that only a small subset of the samples underwent preliminary testing before determining that an additional purification step was required. For potassium dichromate, C_q_ values were significantly improved after the purification step, whereas for other preservation methods the detection of IAC and *N*. *americanus* DNA was feasible only after the inclusion of the purification step.
(XLSX)

S2 Table Analysis of deviance table for GLM at -20°C. This table shows that there was no significant effect on the integrity of the samples over time (*F* = 0.079, p = 0.780) for those samples stored at -20°C.
(XLSX)

S3 Table Analysis of deviance table for GLM at 4°C. At 4°C we fit the full model specified in Eq 1. The marginal significance of the interaction term (F = 1.825; p = 0.083) suggests that there is only weak evidence that these methods differ in their preservation ability over time. That is, at 4°C, the differences in C_q_ are attributable to the application of the preservation methods initially and not to the differences in the performance of these methods over time.
(XLSX)

S4 Table Coefficients from the GLM along with their standard errors (SE) at 32°C. The first column shows the method of preservation employed (indexed by j). In the second column, the value in the first row is exp(α_0_) and the values in all subsequent rows are exp(α_1j_). These are exponentiated coefficients associated with each treatment upon initial application of the preservative (measured by C_q_ values). Each coefficient (except for the first one) shows how much higher than the control/”no preservative” treatment the estimated C_q_ value is, at time zero (t = 0) when that method is employed. FTA cards are 41% higher than the control, which is 15.66 (the standard errors associated with each of the coefficients are in the parentheses). The term “Relative Initial Effect” corresponds to the C_q_ difference relative to the control upon application of the preservative (at time t = 0). The third column shows the 95% confidence interval associated with each of the estimated coefficients in the second column. The fourth column includes the exponentiated coefficients associated with each treatment per day of storage, measured by C_q_ values (the standard errors associated with each of the coefficients are in the parentheses). Each coefficient (except for the first one) shows how much higher the C_q_ value is expected to be for each additional day of storage when this particular treatment is employed. For instance, the coefficient for the FTA cards is 0.9956, but the respective coefficient for the control/”no preservative” treatment is 1.0045, so the overall effect is about 1. This can be interpreted to mean that the influence of FTA card storage effectively offsets the decay of the DNA target seen in the control/”no preservative” treatment. We have coined this the “Relative Daily Effect” which corresponds to the increase in sample C_q_ value relative to the increase in C_q_ value of the control per day of storage. The fifth column shows the confidence intervals associated with each of the estimated coefficients in the fourth column. FTA cards are the only preservation technique for which the initial confidence interval does not include 1.
(XLSX)

S5 Table Mean C_q_ values from the quantitative real-time PCR testing of all *N*. *americanus* biological replicates (n = 9) for each preservative, temperature and time point (total n = 628). (XLSX)
